# Label-free plasmonic nanostar probes to illuminate *in vitro* membrane receptor recognition†
†Electronic supplementary information (ESI) available: Experimental details, Fig. S1–S9, and Table S1. See DOI: 10.1039/c8sc05035j


**DOI:** 10.1039/c8sc05035j

**Published:** 2018-12-04

**Authors:** Sian Sloan-Dennison, Zachary D. Schultz

**Affiliations:** a Department of Chemistry and Biochemistry , The Ohio State University , Columbus , OH 43210 , USA . Email: Schultz.133@osu.edu

## Abstract

Protein–ligand recognition is a key activity where chemical signals are communicated to cells to activate various biochemical pathways, which are important for understanding membrane signaling and drug interactions. Gold nanostars are highly attractive for biological applications due to their readily modified surface chemistry, facile synthesis and optical properties. The increase in electromagnetic field at their branches increases the surface enhanced Raman scattering (SERS) making them ideal candidates as label free *in vitro* probes that can be used to detect a variety of cellular activities. However, the use of particles *in vitro* is complicated by the adsorption of proteins, which forms the protein corona. In this paper we demonstrate gold nanostars as label free *in vitro* probes to study the interaction between α_v_β_3_ integrin and RGD. Nanostars functionalized with cyclic-RDGFC reduced the formation of the protein corona, due to its zwitterionic nature, indicating a small peptide approach to minimizing protein absorption. Additionally, the functionalized nanostars evince a SERS response from their interaction with α_v_β_3_ integrin representative of the amino acids present at the binding site which is also retained in a complex biological matrix. The nanostars were used *in vitro* to selectively detect α_v_β_3_ integrin on the membrane of human metastatic colon cancer cells. By exploiting the intense SERS and tunable plasmon resonance properties of gold nanostars functionalized with cyclic RGDFC, we have demonstrated a label free approach to investigate the chemical interactions associated with protein–ligand binding from both purified proteins and membrane bound receptors in cells.

## Abstract 2

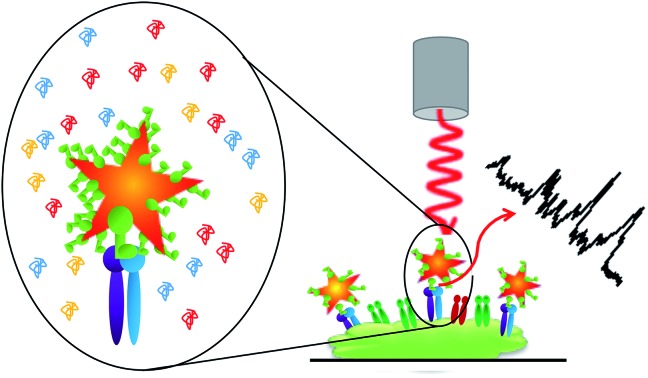
Peptide functionalized plasmonic nanostars evince Raman signals from targeted receptors in cells and modulate protein corona formation, improving targeting.

## Introduction

Nanomedicine promises to leverage the unique properties of nanoparticles (NPs) to target and treat disease; yet complications associated with low delivery efficiency have stalled their use in clinical practice.^1^ Plasmonic NPs are widely used for site selective targeting and tagging in imaging studies of cell membrane receptors due to their attractive properties such as size, charge and ease of chemical functionalization. Systems have been developed and implemented to target cellular components with ligands for imaging and drug delivery,^2^–^4^ providing information about the molecular level interactions that occur between the ligand and receptor, an important factor in our understanding biochemical signaling and specificity related to drug interactions.^5^

In all these applications, targeting is based on the selectivity of the attached biorecognition agent, however this can be hindered by the environment the NPs are suspended in due to the formation of the protein corona.^6^–^8^ When NPs are incubated with cells or in other biological fluids, the adsorption of serum proteins and biomolecules often results in the formation of a protein corona, which can be characterized as hard or soft to reflect the strength of adhesion to the NP.^9^ When proteins bind to the surface of the NP *via* van der Waals, hydrogen bonding, hydrophobic, electrostatic and π–π interaction, a hard protein corona is formed.^10^ Loosely associated and rapidly exchanging layers of proteins produce the soft corona where proteins have lower dissociation constants and are readily desorbed.^11^

Although the protein corona can facilitate the interaction of NPs and cells, the formation of a hard corona will also dictate the NP distribution, macrophage uptake and can interfere with targeting molecules on the NPs by obscuring their binding region.^12^ The hard corona can also be detrimental to the NP itself, leading to aggregation and change in shape, charge and scattering properties. Fortunately, NPs can be functionalized with short oligo (ethylene glycol) spacers with a range of termini^13^ or cysteine,^14^ to create zwitterionic coatings producing ‘corona free’ NPs which only experience the reversibly bound soft protein corona. Furthermore, when cysteine and a targeting molecule were functionalized to the surface of silica coated gold NP, the conjugates were able to inhibit corona-induced mistargeting and thus significantly enhance the active targeting capability of NPs in complex biological media. It is therefore vitally important to characterize the formation and if possibly eliminate the hard protein corona to be able to properly classify signals observed from *in vitro* cellular experiments.

NPs are commonly detected by imaging, or mapping, the cell with an appropriate laser excitation and monitoring the spectral response of the NP. Excitation of plasmon resonances has been correlated with enhanced Raman signals, an effect commonly referred to as surface enhanced Raman scattering (SERS).^15^ SERS is increasingly used to image the location of cellular proteins.^16^ For example, silver (Ag) NPs and silica encapsulated hollow gold (Au) nanospheres that have been coated in organic Raman labels and antibodies are used to detect the cancer targets HER2 and CD10 and quantify breast cancer phenotypic markers expressed on cell surfaces.^17^,^18^ While these examples provide image contrast, the Raman response of cellular components can also be enhanced to provide chemical information from biomolecules interacting with the NPs.^19^ While typically much lower intensity than reporter molecules attached to the NPs, the SERS signal of cellular components is reported to provide chemically specific biomolecular characterization.

Integrins are a class of transmembrane cell adhesion proteins that interact with proteins in the extracellular matrix to activate intracellular pathways depending on the character of the extracellular stimuli.^20^ α_v_β_3_ integrin is highly expressed on activated cells making it a common drug target, as inhibiting its signaling has potential for the treatment of cancer.^21^ α_v_β_3_ integrin has an affinity for the peptide chain arginine–glycine–aspartic acid (RGD) which has routinely been used to coat the surface of Au NPs to target α_v_β_3_ integrin on activated cells. Typically Raman^22^ or fluorescent labels^23^ are incorporated to image the cells, and the chemical information that could be gained from the RGD ligand and α_v_β_3_ integrin binding is lost. To preserve this information, label free NP probes need to be developed. Spherical NPs are capable of providing SERS enhancement of Raman labels, but the detection of native biomolecules in cells can be challenging. Recent work has shown that the signal of NPs binding to integrin proteins can be selectively detected using tip-enhanced Raman scattering (TERS).^24^–^26^ The selective detection has been attributed to the coupling between the TERS tip and the spherical NP probe,^24^ where the coupled structures exhibit a stronger plasmonic effect than either the tip or NP alone. Asymmetric NPs often exhibit larger plasmon associated fields, suggesting an alternative approach.^27^

One class of asymmetric NP with particular promise is nanostars (NSs), which are multi-branched particles that, upon plasmonic excitation, can generate enormous electromagnetic fields at their vertices, providing SERS hotspots without aggregation.^28^ The localized surface plasmon resonance (LSPR) of NSs can be manipulated to match the excitation laser wavelength, which generates on resonance Raman scattering to further increase the signal observed.^29^ NSs produce a tremendous SERS enhancement of Raman labels and this methodology has been utilized *in vitro* to target and image cells.^30^ The increased SERS signals from NSs also makes them desirable label free probes. For instance, Ag NS-patterned substrates functionalized with RGD peptides have been utilized for cellular analysis. Cells were added to the substrate and imaged using 785 nm laser excitation which gave biochemical characterization of breast cancer cells.^31^ Also, He *et al.* have demonstrated a label free approach for the detection of protein kinase A (PKA), a potential prognostic marker and predictor of prostate cancer by using Au NSs functionalized with kemptide.^32^ When PKA was added to Au-kemptide NS, phosphorylation of the kemptide occurred that significantly altered the SERS signal, indicating the presence PKA.

In this paper, we demonstrate the use of functionalized Au NSs as label free probes of membrane receptors. By functionalizing Au NSs with cyclic RGDFC and incubating with purified α_v_β_3_ integrin, the SERS spectra of the resulting conjugates can be analyzed using multivariate curve resolution to generate a characteristic SERS spectrum of RGDFC–α_v_β_3_ integrin interaction. The RGD targeting is retained even after incubation with serum proteins, and characterization suggests that RGDFC coating eliminates the formation of the hard corona allowing them to be used as selective *in vitro* probes. Using the SERS spectrum from the purified receptor, we show RGDFC functionalized Au NSs can selectively target and image α_v_β_3_ integrin on the membrane of a human metastatic colon cancer cell line.

## Results and discussion

### Nanostar synthesis and functionalization

Gold nanostars (Au NSs) were investigated as a label free probe to characterize ligand binding to protein receptors in the plasma membrane of cells. The Au NSs were synthesized using a seed mediated method adapted from that reported by Zhang *et al.*^33^ Experimental details are provided in the ESI.† The extinction spectra and SEM images of the resulting NSs are shown Fig. S1 (ESI†). The Au NSs with an LSPR of 730 nm were functionalized with a final concentration of 200 μM of cyclic RGDFC peptide, whose structure is shown in Fig. S2 (ESI†). Cyclic RGDFC contains the peptide motif RGD, which is reported to selectively bind to α_v_β_3_ integrin.^34^ The cystine (C) residue provides a thiol for easy functionalization. Additionally, the phenylalanine (F) gives a distinct Raman band at 1000 cm^–1^ in the SERS spectrum, which was used to confirm successful binding to the NS. Upon functionalization the Au and Au-RGDFC NSs were characterized using extinction spectroscopy and SERS, as shown in Fig. S3 (ESI†).

The extinction spectra of Au-RGDFC NSs indicate the LSPR of the functionalized NSs are blue shifted with respect to bare Au NSs. Particles are known to modify their shape to minimize the surface energy *via* the Ostwald ripening effect.^35^ The blue shift is consistent with the branches collapsing into less sharp and elongated protrusions. Despite this change in shape, the particles still exhibit SERS enhancement of the RGDFC ligand, as evident by the clear 1000 cm^–1^ phenylalanine peak and 1030 cm ^–1^ arginine peak. In contrast, the Au NS SERS spectra only displays SERS signals associated with the surfactant CTAC.

### α_v_β_3_ integrin binding

Recently, we demonstrated that TERS can be used to investigate the interaction between protein and ligands by selectively detecting the Raman signals of integrin from not only the purified receptor but from intact cell membranes as well.^26^,^36^ By utilizing Au-RGDFC NPs, which bound to the corresponding integrin on a cell membrane, plasmonic coupling between a spherical Au NP and the TERS tip produced a strong characteristic TERS signal of the bound integrin. Interestingly, the high aspect ratio branches of the NS are similar in structure to the NP interacting with a TERS tip. Although TERS offers very high spatial resolution as well as potential single molecule detection,^37^ TERS is a complicated experiment; only small areas of a cell can be interrogated at a time, and issues can occur due to the limited availability of quality TERS tips.^38^ Au NSs offer the possibility to assess entire cells quickly. To address these issues, Au NSs with vertices and branches that resemble the TERS tip-nanoparticle construct that generate large Raman enhancements are investigated as an alternative.

**Fig. 1:**
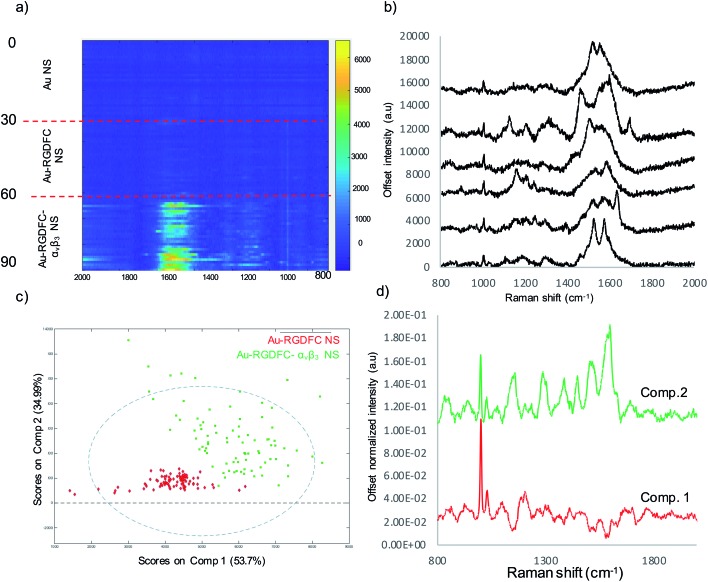
(a) Heat map constructed from baseline corrected SERS spectra of Au, Au-RGDFC and Au-RGDFC–α_v_β_3_ NS (*n* = 30, each), (b) selected SERS spectra from the Au-RGDFC–α_v_β_3_ NS map, (c) MCR scores plot of SERS data from Au-RGDFC (red) and Au-RGDFC–α_v_β_3_ NS (green) and (d) loading plots of MC1 (red) and 2 (green).

Fig. 1 shows the SERS results when Au-RGDFC NSs were combined with purified α_v_β_3_ integrins. Au-RGDFC NSs and α_v_β_3_ integrin were incubated together for 2 hours to allow binding between the peptide and integrin to occur, Fig. 1a is a heat map constructed from 30 spectra acquired from each of the Au, Au-RGDFC and Au-RGDFC–α_v_β_3_ NS conjugates (90 spectra in total). New peaks are observed upon the addition of the integrin, which highlight the enhancement due to the bound α_v_β_3_ integrin. Selected spectra of the Au-RGDFC–α_v_β_3_ NSs are shown in Fig. 1b illustrating the level of variation observed in the SERS spectra. This variation likely arises from changes in the protein orientation affecting the parts of the macromolecule which experiences the SERS enhancement.^39^

Chemometric analysis was used to analyze the spectral differences between Au-RGDFC and Au-RGDFC–α_v_β_3_ NSs. The SERS data set (180 spectra in total, 90 from each samples) were analyzed using multivariate curve resolution (MCR), a method that analyses the variance in data and produces components associated with the spectral composition. The MCR plot is shown in Fig. 1c and many of the data points from both samples (Au-RGDFC NS, red and Au-RGDFC–α_v_β_3_ NS, green) are grouped on component 1. The loading plot of MC1 indicates this is due to RGDFC spectral composition, particularly the prominent 1000 cm^–1^ peak, and closely resembles the average SERS spectrum of Au-RGDFC NS. The data points on component 2 are attributed to Au-RGDFC–α_v_β_3_ NSs and arise from the enhanced Raman scattering of the amino acids found at the binding site between RGD and α_v_β_3_ integrin. The peaks from the α_v_β_3_ integrin generated from MCR component 2 loading plot were assigned using previous TERS studies and included phenylalanine (1000, 1586 cm^–1^), lysine (1078 cm^–1^),C–H bending (1288, 1446 cm^–1^), and tryptophan (1386, 1586 cm^–1^).^25^,^26^ More peak assignments can be found in Table S1 of the ESI.† Component 2 thus provides a SERS signature that can be used to evaluate spectra observed in cells.

### Protein corona characterization

**Fig. 2:**
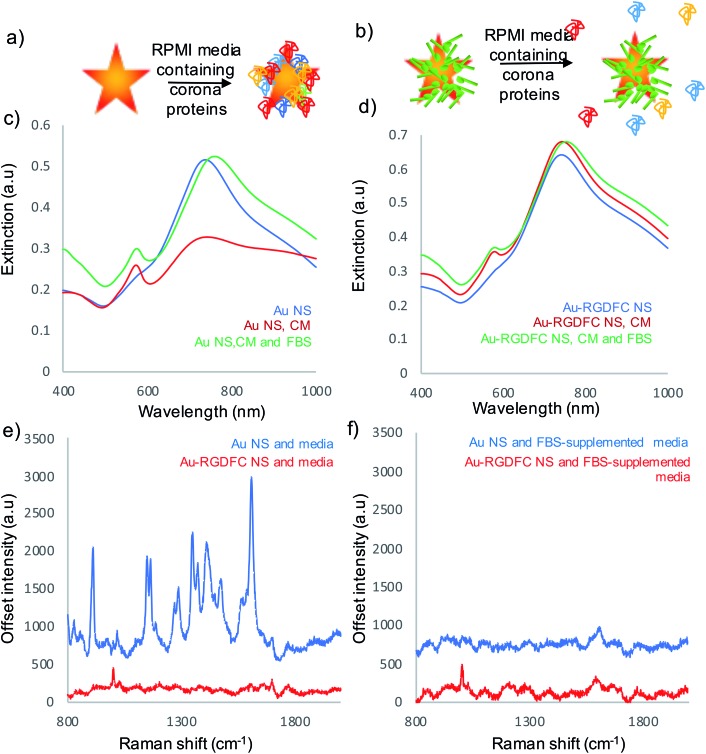
(a) Schematic of protein corona formation on bare Au NS when incubated with media and (b) the lack of protein corona formation on Au-RGDFC NS when incubated with media. (c) Extinction spectra of Au NS and (d) Au-RGDFC NS without (blue) and with cell media (red) and FBS supplemented cell media (green), (e) SERS spectra of Au NS (blue) and Au-RGDFC NS (red) after incubation with cell media and (f) Au NS (blue) and Au-RGDFC NS (red) after incubation with FBS supplemented media. The SERS spectra are the average from 10 nanoparticles clusters in the focus of a 50× microscope objective, excited using 0.8 mW of 633 nm laser excitation, and scanning between 800–2000 cm^–1^.

Experiments were performed to assess the impact of protein corona formation on the Au NS probes. Au and Au-RGDFC NS were incubated with RPMI media and fetal bovine serum (FBS) supplemented RPMI media for 2 hours. The resulting Au NSs were characterized by extinction spectroscopy, size measurements and SERS using 633 nm laser excitation. A schematic of the protein corona formation on bare Au NSs and Au-RGDFC NSs is shown in Fig. 2a and b and associated extinction and SERS spectra shown in Fig. 2c–f. Table 1 indicates the change in size before and after incubation with media.

**Table 1:** Change in size after incubation with media and FBS supplemented media

NS sample	Before (nm)	Incubated with RPMI media (nm)	Incubated with FBS supplemented RPMI media (nm)
NS	138.4 ± 1.9	199 ± 2.16	478 ± 43.6
NS-RGDFC	141 ± 12.1	157 ± 5.1	179 ± 3.6

RPMI media consists of many amino acids such as tyrosine, tryptophan, histidine and arginine^40^ which electrostatically adsorb to the bare Au NS surface leading to aggregation, indicated in Table 1 and Fig. 2c (red spectrum) and their appearance in the SERS spectrum, Fig. 2e (blue). There was little increase in size and change in extinction spectra, Fig. 2d (red spectrum) observed when Au-RGDFC NSs were incubated with RPMI media and the observed SERS spectrum, Fig. 2e (red), arose only from the RGDFC. The lack of new amino acid signals with RGDFC functionalized NS indicates that functionalization with a high concentration of RGDFC inhibited their adsorption, adding a protective layer to the NS.

FBS is added to RPMI media before incubation with cells due to its low abundance of antibodies and high content of embryonic of growth factors giving it ideal properties for survival and growth of cells during cell culture.^41^ The major component of FBS is the large protein bovine serum albumin (BSA) which is 66.4 kDa.^42^ Low concentrations of BSA can be used to coat NPs protecting against aggregation and show little hemolysis and cytotoxic response;^43^ however, the high concentration in the FBS supplemented RPMI media forms a protein corona along with the other serum proteins when incubated with Au NSs. Our results show incubating the Au NSs with FBS supplemented media leads to a massive increase in size (478 nm) and a large shift in the LSPR (30 nm), Fig. 2c (green), evidence that a hard protein corona has formed. The protein corona formation cannot be analyzed with SERS as it has poor Raman properties, Fig. 2f (blue). The lack of peaks indicates that the protein corona formed by the addition of FBS resulted in complete monolayer coverage of the NS, inhibiting the adsorption of other cell media components. The hard protein corona formation significantly changed the bio-identity of the NS, reinforcing the need for a protective layer.^11^

A more desirable outcome was achieved when Au-RGDFC NSs were incubated with FBS-supplemented RPMI media. A small LSPR shift (13 nm), Fig. 2d (green), and an increase in size of 28 nm was obtained. The FBS supplemented media also had little effect on the RGDFC which is still visible in the SERS spectrum after incubation, Fig. 2f (red). The absence of amino acid signals after incubation suggested that RGD does not bind to any substituents of the RPMI media which is consistent with previous experiments that show Au-RGDFC NP will selectively bind to the integrin receptors.^25^ Further analysis indicated that the size of the components in the FBS supplemented cell media were roughly 30 nm in diameter, the same size as the increase in size on the Au-RGDFC NS after incubation. This suggested that a small number of serum proteins were attracted to the Au-RGDFC NS and it was proposed that they formed a soft protein corona over time. Soft protein coronas consist of reversible interactions and are less detrimental as the proteins do not bind to the surface of the NS, therefore not affecting the size, charge or targeting of the probes.^14^

It is interesting that the RGD coating appears to inhibit the formation of the hard protein corona. Typically, BSA or high density layers of polyethyleneglycol (PEG) are grafted to NPs to inhibit protein adsorption.^11^ However, there has been a number of recent studies that have reported that functionalizing a zwitterionic surface to the NP surface will reduce the formation of the protein corona.^13^,^14^,^44^ Therefore, it is not unusual that the RGDFC peptide, which has both a positive (arginine) and negative (glutamic acid) residue, exhibits reduced formation of the hard protein corona. Another recent report indicates that the surface chemistry is the critical factor for controlling the fate of nanoparticles in biological systems.^45^ Surface charge density has also been suggested to control protein absorption.^46^ The charge on the surface has been disputed as the determining factor due to the short Debye screening lengths under physiological conditions.^47^ We speculate that the RGD coating is not conducive to binding by the cell media proteins as this would inhibit transport to cells in living organisms. This suggests that other biomimetic functionalization's may also be effective at inhibiting unwanted protein adsorption. In fact, the protein corona has been used deliberately to alter targeting of nanoparticles and for drug delivery.^48^

**Fig. 3:**
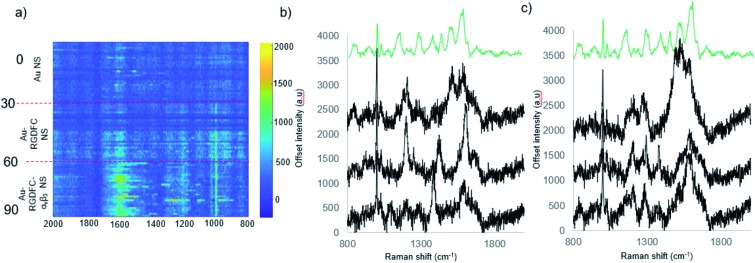
(a) Heat map constructed from baseline corrected SERS spectra of Au, Au-RGDFC and Au-RGDFC–α_v_β_3_ NSs incubated with FBS supplemented RPMI cell media (*n* = 30, each). (b) and (c) Selected spectra of Au-RGDFC–α_v_β_3_ NS (black) and MCR spectra of purified α_v_β_3_ integrin (green).

To investigate if the soft protein corona formation on Au-RGDFC NS affected the probes targeting properties, Au-RGDFC NS were incubated with FBS supplemented RPMI media and α_v_β_3_ integrin for 2 hours and analyzed using 633 nm laser excitation. The resulting SERS spectra was compared to Au and Au-RGDFC NS incubated with FBS supplemented RPMI media shown in Fig. 3a.

The heat map in Fig. 3a indicates an increase the number of peaks and their intensity when Au-RGDFC NS were incubated with FBS supplemented media and the α_v_β_3_ integrin. Selected spectra from the heat map are shown in Fig. 3b and c and many of the enhanced peaks have similar Raman shifts as the MCR generated SERS spectrum of α_v_β_3_ integrin including phenylalanine and tryptophan at 1591 cm^–1^, and 1207 cm^–1^, C–C and C=C at 1511 cm^–1^ and C–H bond at 1289 cm^–1^. The results indicate that despite the formation of the soft protein corona, the interactions involved are relatively weak and reversible, such that RGD is still able to bind to the α_v_β_3_ integrin. The observed SERS signals most likely arise due to the α_v_β_3_ integrins amino acids that are at the binding site and agree with our previous reports.^24^ The results suggest that Au-RGDFC NS can be used as *in vitro* probes of protein recognition.

### *In vitro* ligand-receptor binding characterization

To assess Au-RGDFC NS targeting of α_v_β_3_ integrin's in intact cell membranes, Au-RGDFC NSs were incubated with SW620 colon cancer cells *in vitro*. Prior to incubation, the Au NS size (138 nm), zeta potential (33 mV) and concentration (2.99 × 10^10^ particles per mL) were calculated. The NSs were added into the culture dish for 2 hours and the cells were then fixed before SERS measurements. Bare Au NSs were added to separate SW620 cells as a control sample. The cell membrane was brought into focus using a 50× objective lens and individual cells were mapped with a 1 μm step by Raman spectroscopy using 633 nm laser excitation.

**Fig. 4:**
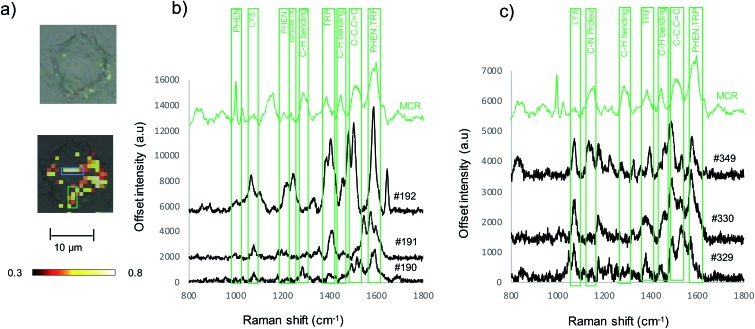
(a) White light and false color image of SW620 cell incubated with Au-RGDFC NS created using the MCR generated spectrum of purified α_v_β_3_ integrin as a reference. Cells were mapped using a 50× microscope objective, 1 × 1 μm step size, 633 nm laser excitation with a laser power of 4 mW, scanning between 800–1800 cm^–1^. MCR reference spectrum (green) and extracted spectra from within the blue box (#190, 191 and 192) (b) and from within the green box (#329, 330, 349) (c).

Previous TERS results indicate the α_v_β_3_ integrin was selectively enhanced on a cell membrane. To examine if this selectivity is maintained using Au-RGDFC NSs, Raman maps were acquired and analyzed. The spectra from the resulting Raman maps were analyzed with direct classical least squares (DCLS) using the WiRE software and the reference spectrum of purified α_v_β_3_ integrin generated from the MCR analysis (Fig. 1d). Fig. 4a shows the white light and false color image of a cell incubated with Au-RGDFC NSs. The false color image, created from DCLS analysis with Renishaw WiRE software, indicates areas where the spectra matches the MCR spectrum of purified α_v_β_3_ integrin. Select spectra from the cells within the blue (Fig. 4b) and green (Fig. 4c) box are shown and the observed peaks show agreement with peaks in the reference spectrum of the α_v_β_3_ integrin. This suggests that the Au-RGDFC NSs attach to the surface of the cell membrane due to RGD recognizing the α_v_β_3_ integrin and strong SERS signals of the bound α_v_β_3_ integrin are observed. As expected, due to the large size of the NS, other components of the cell membrane, such as phospholipids, may also be enhanced as they are near the site of RGD–α_v_β_3_ integrin binding. The α_v_β_3_ integrin signal is not uniform on the cell and Au-RGDFC NSs appear to aggregate on the cell membranes resulting in localized areas of intense SERS spectra. This is consistent with previous reports of integrin clustering which has been modelled and is controlled by ligand density and other mechanical properties of the extra cellular matrix.^49^ Fig. S4 (ESI†) reports replicated data of the DCLS false color image and selected spectra obtained from Au-RGDFC NS incubated with cells demonstrating reproducibility.

**Fig. 5:**
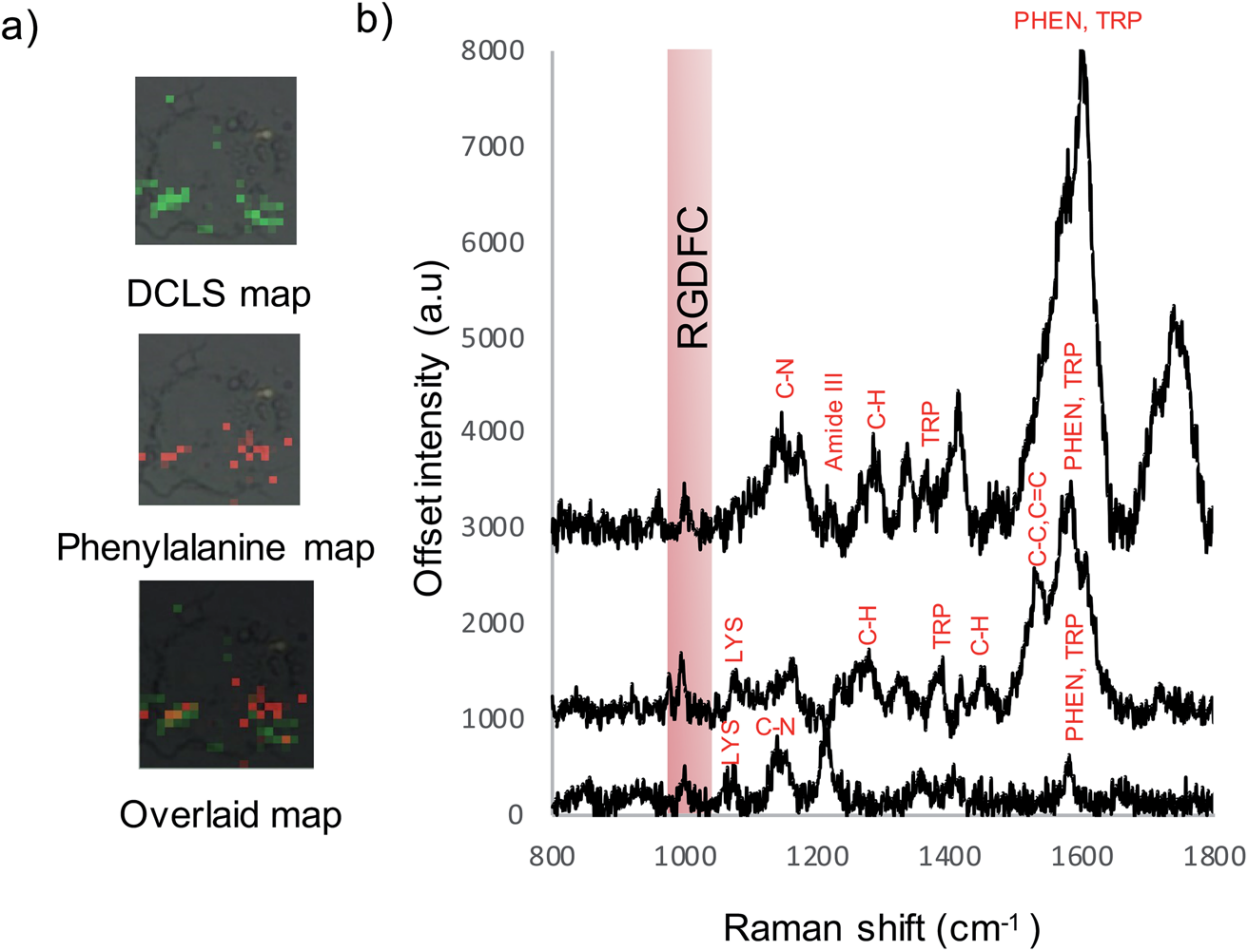
(a) False color images created using DCLS analysis, RGDFC peak at 1000 cm^–1^ analysis and the two overlaid indicating areas of overlap. (b) Selected spectra from map indicating RGDFC peaks and peaks of the bound α_v_β_3_ integrin spectrum. Cells were mapped using a 50× microscope objective, 1 × 1 μm step size, 633 nm laser excitation with a laser power of 4 mW, scanning between 800–2000 cm^–1^.

As cell membranes are made up of many different components, phospholipids, carbohydrate groups, *etc.*, the cell maps were evaluated further to confirm that the signal originated from the bound α_v_β_3_ integrin and that the RGDFC peptide was not binding to other membrane components. False color images were created by monitoring the main peak of the RGDFC peptide (1000 cm^–1^ peak of phenylalanine) shown in Fig. 5a. Comparison with the DCLS false color image, which indicates the area of high spectral matches to the MCR α_v_β_3_ integrin spectrum, shows both images have the same highlighted areas of the cell (overlaid image) suggesting that Au-RGDFC NS are only binding with a high affinity for α_v_β_3_ integrin. Three spectra from the overlapped area indicate the presence of the RGDFC ligand which always coincides with peaks of bound α_v_β_3_ integrin, shown in Fig. 5b. This result agrees with our previous reports.^25^ It should be noted that the DCLS also highlights areas of the cell with no RGDFC signal which is unexpected as it is thought that it should always be present as it is bound to the NS. However, changes in confirmation of the peptide can occur when the RGDFC binds to α_v_β_3_ integrin such that the phenylalanine is in a different orientation, no longer experiencing the same enhancement.

**Fig. 6:**
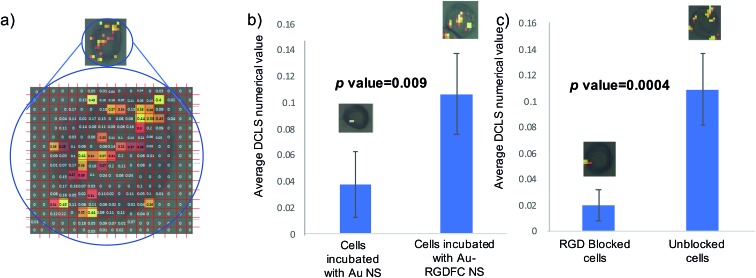
(a) Numerical analysis of NS incubated cells. A false color image using DCLS was created and a number was generated for every pixel, indicating how closely it matched the reference α_v_β_3_ integrin spectrum. (b) The bar chart shows the average DCLS numerical value for Au and Au-RGDFC NS incubated cells. (c) The bar chart reports the average DCLS value when cells are incubated with free RGD in solution (blocked) *versus* unblocked cells. Error bars are the standard deviation from 5 cells. *T*-tests performed on the data provided the indicated *p*-values.

To test the hypothesis that specific binding between the RGDFC and α_v_β_3_ integrin brings the NS to the cell surface and provides the SERS enhancement and not electrostatic interactions between the positively charged NS and negative cell membrane components, cells were incubated with bare Au NS and analyzed. False color images were created using DCLS with Renishaw WiRE software and the resulting images of 5 Au NS incubated cells and 5 Au-RGDFC NS incubated cells were compared, Fig. S5 (ESI†). The images indicate a clear difference in the number of spectra which have similar spectral contributions to the MCR generated spectrum of α_v_β_3_ integrin due to the decrease in the number of colored pixels in the Au NS incubated cell. Another difference to note is the appearance of signals outside the cells which occurs with the Au NS samples, which is characteristic of the NS aggregating on the glass surface and therefore only enhancing the components from the RPMI media. To place a numerical value on this difference, the spectra from the map were assigned a ‘DCLS comparison number’ which represented how closely it matched the MCR α_v_β_3_ integrin spectrum. Fig. 6a illustrates how the maps were processed. Each pixel represents an individual spectrum and the number relating to how closely is resembles the α_v_β_3_ integrin spectrum is shown in the middle. To assign a numerical value to the whole cell, the average of all pixels within the map was taken and the bar chart in Fig. 6b is the average DCLS comparison number accumulated from 5 different cells incubated with Au or Au-RGDFC NSs. Comparing the average of each sample, it is evident that a larger number of spectra from the cells incubated with Au-RGDFC NSs have characteristic α_v_β_3_ integrin features, reinforcing that the Au-RGDFC NSs are binding to α_v_β_3_ integrin. The observation of peaks not characteristic of α_v_β_3_ integrin suggests electrostatic interactions can occur and enhance other components of the membrane, but to a much lower extent. The biological variability results in some uncertainty; however, analysis shows the results are statistically significant (*p* value = 0.009).

The SERS enhancement offers clear advantages for understanding protein–ligand interactions. By selectively detecting α_v_β_3_ integrin on the surface of SW620 cells without the use of a Raman label a wealth of information such as the location, clustering and proteins involved in the binding is acquired. Additionally, Raman signals arising from the amino acids in the protein are observed. Previous work shows the amino acids involved in binding to the protein are selectively enhanced.^25^ The variance in the observed spectrum has been reported as a measure of binding specificity and the observed Raman bands inform on the amino acids at the binding site. This vital data is important in understanding biochemical signaling and drug interactions, creating a novel method for detecting the interactions between small ligands and protein receptor binding.

To further validate the selectivity of this approach, receptor blocking experiments were also performed on the cells. The α_v_β_3_ integrin on SW620 cells were blocked by incubating the cells with a high concentration of the linear RGD peptides for 2 hours, which is sufficient time to allow the binding between the two to occur. Au-RGDFC NSs were then added to the blocked cells and unblocked cells (acting as a control). The NS were incubated with the cells for 2 hours before being washed and fixed. The resulting cells were mapped using 633 nm laser excitation and false color DCLS images were created as described before and are shown in Fig. S6 (ESI†). As expected little to no signal was acquired when the α_v_β_3_ integrin of the cell was blocked as there were no available sites for the RGD functionalized to the Au NS to bind too. Due to insufficient binding, the NS were washed away and did not provide any enhancement of the α_v_β_3_ integrin or other cellular components. Some control cells still yielded a SERS signal. As described before, a numerical value on how closely the spectra of the cell map matched the α_v_β_3_ integrin was calculated and Fig. 6c is the bar chart clearly showing that the blocked cells have a very low number of spectra which have similar features to the α_v_β_3_ integrin when compared to unblocked cells.

The results presented here illustrate the use of plasmonic NSs to monitor protein ligand interactions *in vitro*. These results indicate new opportunities for assessing nanomedicines and early stage screening of drug targeting prior to advancing to animal models. Specifically, this methodology has potential for early stage drug screening. The SERS spectrum identifies what the ligand on the NS is interacting with, and high-throughput analysis of NSs on cells, and potentially in animal models, may identify potential off-target effects to facilitate drug trials.

## Conclusion

In conclusion, we have demonstrated Au NS can be used to investigate the binding between RGD and α_v_β_3_ integrin. Au NS provided a suitable SERS surface that allowed the amino acids of α_v_β_3_ integrin present at the RGD binding site to be enhanced and a characteristic SERS spectrum of purified α_v_β_3_ integrin was generated using MCR. The detection of α_v_β_3_ integrin was retained even when a complex matrix was added to the solution, FBS supplemented cell media, as the RGDFC coating prevented the formation of a hard corona, which could interfere with targeting agents. The α_v_β_3_ integrin spectrum could then be detected from the surface of colon cancer cells when Au-RGDFC NS were incubated due to the binding between RGD and transmembrane α_v_β_3_ integrin, verifying the receptor interacting with the NS probe. The RGDFC functionalization was demonstrated to prevent the formation of the protein corona *in vitro*. Statistical analysis indicated areas where α_v_β_3_ integrin clustering occurs and the SERS signal of the integrated receptor was not obscured by the protein corona or other non-specific interactions. Blocking experiments confirmed the selectivity of this approach as RGD blocked cells yielded no signal as all α_v_β_3_ integrin binding sites were occupied. The use of functionalized Au NS represents a new method of investigating small ligand-protein binding in intact cell membranes. Possibilities include targeting vascular endothelial growth factor receptors, which could greatly benefit drug development by specifying chemical interactions with potential drug targets.

## Conflicts of interest

There are no conflicts of interest to declare.

## Supplementary Material

Supplementary information
