# Supplementary material for: Label-free plasmonic nanostar probes to illuminate in vitro membrane receptor recognition
Source: Chem Sci. 2018 Dec 4;10(6):1807–15. doi: 10.1039/c8sc05035j (PMC6369441; doi:10.1039/c8sc05035j)
Supplement: Supplementary file 1 [file SC-010-C8SC05035J-s001.pdf]

Electronic Supplementary Material (ESI) for Chemical Science.
This journal is © The Royal Society of Chemistry 2018

Label-Free Plasmonic Nanostar Probes to Illuminate In Vitro Membrane Receptor Recognition

Sian Sloan-Dennison and Zachary D. Schultz*

Department of Chemistry and Biochemistry, The Ohio State University, Columbus, OH 43210

*corresponding author email: Schultz.133@osu.edu

Supplementary information

Table of contents

Material and Methods……………………………………………………………………………………………………2

Supplementary Figures S1-S9…………………………………………………………………………………………4

Supplementary table………………………………………………………………………………………………………..9

References……..9

Materials and Methods

Chemicals

Tetrachloroauric (III) acid ($HAuCl_4$), cetyltrimethylammonium chloride (CTAC), sodium borohydride ($NaBH_4$), silver nitrate ($AgNO_3$), hydrochloric acid (HCl), ascorbic acid ($C_6H_8O_6$) and HPLC grade water were all purchased from Sigma Aldrich (USA). Cyclic(-Arg-Gly-Asp-D-Phe-Cys) purchased from Peptide international. Recombinant human $\alpha_v\beta_3$ integrin protein ($\alpha_v\beta_3$) was purchased from R&D systems. All cell culture reagents were purchased from Thermo Fischer Scientific (USA).

Instrumentation

Raman measurements were carried out using a Renishaw InVia instrument with 633 nm laser excitation. Extinction measurements were carried out using a VWR V-1600 Spectrophotometer. Scanning electron microscope (SEM) images were obtained using a Magellan XHR SEM. Size and zeta-potential measurements were carried out using a Malvern Zetasizer ZS and concentration calculated using a Malvern Nanosight 300NS.

Au NS Synthesis

Au multi-twined seeds were prepared by adding $HAuCl_4$ (50 µl, 10 mM) to CTAC (1 mL, 100 mM). This solution was mixed for 30 minutes whilst $NaBH_4$ solution (10 mM) was cooled in a 4 °C fridge. After 30 minutes, 150 µL of cold $NaBH_4$ was added to the solution and left to mix for an additional 3 minutes. Upon addition of the chilled $NaBH_4$, the solution turned orange signifying the synthesis of Au multi-twined seeds and was left overnight in the fridge prior to use. To grow the NS, a CTAC solution (10 mL, 100 mM) was prepared and 1 mL was removed and added to 1 µL of seed. The growth solution was prepared by adding $HAuCl_4$ (450µL, 10 mM), $AgNO_3$ (90 µL, 10 mM), HCl (180 µL, 1 M) and $C_6H_8O_6$ (90 µL, 100 mM) to the remaining CTAC solution and left to mix for 5 minutes. The growth solution was split into 9x1 mL vials and different volumes of seed solution were added to each vial (800, 600, 400, 200, 100, 50, 30, 10, 1 µL). The solutions were left to shake for 1 hour. Then solutions were centrifuged at 6000 RPM for 20 minutes, and then resuspended in 1 mL of HPLC grade water (H_2O) to remove excess CTAC. The centrifugation and resuspension were repeated twice. The NS were characterized using extinction spectroscopy and SEM. The chosen NS that were incubated with cells were also characterized for size, zeta potential, and concentration using a NanoSight (Malvern, Zetasizer ZS (Malvern) and extinction by UV-Vis spectroscopy.

SERS of RGDFC

Cyclic RGDFC (200 µM) was added to 1 mL of Au NS with LSPRs of 720 nm and left to incubate overnight. The sample was then centrifuged and resuspended (as described before) to remove excess RGDFC. The extinction of the Au-RGDFC NS was taken and compared to Au NS. Au and Au-RGDFC NS were then concentrated to a volume of 100 µL, from which 2 µL was spotted onto a clean glass slide (washed three time with ethanol and H_2O) and left to dry. Average SERS spectra were obtained by focusing on 10 different particle clusters using a 50x objective lens and interrogated with 633 nm laser excitation with a power of 0.8 mW, centered at 1000 $cm^{-1.}$

SERS of $\alpha_v\beta_3$ integrin

$\alpha_v\beta_3$ integrin (2 µL of 200 µg/mL) was added to 10 µL of Au-RGDFC NS and left to incubate for 2 hours. 2 µL of Au-RGDFC- $\alpha_v\beta_3$ NS conjugates were spotted onto a clean glass slide and left to dry. Spectra were obtained by focusing on different particle clusters with a 50x objective lens and interrogated with 633 nm laser excitation, with power of 0.8 mW, scanning between Raman shifts of 800-2000 cm^{-1}. Raman analysis was also carried out for Au and Au-RGDFC NS.

Characterization of protein corona

10 µL of Au or Au-RGDFC NSs were added to 10 µL of RPMI cell media or 10 µL of RPMI media supplemented with 10% FBS and incubated for 2 hours. 2 µL of the conjugates were then spotted onto a clean glass slide and left to dry. Average spectra were obtained by focusing on different particle clusters with a 50x objective lens and interrogated with 633 nm laser excitation, with power of 0.8 mW, scanning between Raman shifts of 800-2000 cm^{-1}. Extinction and size measurements were carried out by adding 500 µL of Au or Au-RGDFC NS with 500 µL of RPMI cell media with and without the addition of 10% FBS and analyzed after incubating for 2 hours.

SERS of $\alpha_v\beta_3$ integrin in complex matrix

10 µL of Au-RGDFC NSs, 10 µL of FBS supplemented RPMI media and 2 µL of $\alpha_v\beta_3$ integrin (200 µg/mL) were added together and incubated for 2 hours. FBS supplemented media was also added to Au and Au-RGDFC NS. 2 µL of all the conjugates were spotted onto a clean glass slide and left to dry. Spectra were obtained by focusing on different particle clusters with a 50x objective lens and interrogated with 633 nm laser excitation, with power of 0.8 mW, scanning between Raman shifts of 800-2000 cm^{-1}.

NS cell incubation and SERS analysis

SW620 cells were cultured in FBS supplemented RPMI media using previously reported published procedures [1] and cells and media transferred to a culture flask (2 mL) with coverslip. 200 µL of Au NS (acting as a control) and Au-RGDFC NS were added to the culture flask and incubated for 2 hours. The cell media was then removed and the cells which had adhered to the coverslip were washed with 2 mL of sterile PBS (three times) and fixed by immersing the coverslip in 4% formaldehyde for 15 minutes. The coverslip was then removed and washed again with PBS and H_2O and left to dry. The cells were then imaged by focusing on the cell membrane with a 50x laser objective with a numerical aperture of 0.75 and Raman mapped using a 1x1 µm step size, 633 nm laser excitation, with a laser power of 4 mW, scanning between 800-2000 cm^{-1}.

RGD Blocking

2 mL of FBS supplemented RPMI media and SW620 cells were added to a culture flask with coverslip. 200 µL of RGD (stock 7.3 mM) was added to flask and left incubating for 2 hours. Au-RGDFC NS were the added to the flask for an additional 2 hours and the cells were washed, fixed and analyzed as described in the previous section.

Data analysis

Raw SERS spectra were preprocessed (baselined and normalized) prior to analysis using the PLS toolbox plugin. Multivariate curve resolution (MCR) was carried out to classify the SERS. Heat and score maps were reconstructed using MATLAB. False color images were created using WiRE software on baselined SERS maps (baselined with WiRE intelligent fit) using direct classical least squares (DCLS) with the MCR generated $\alpha_v\beta_3$ integrin spectrum as a reference.

Supplementary Figures

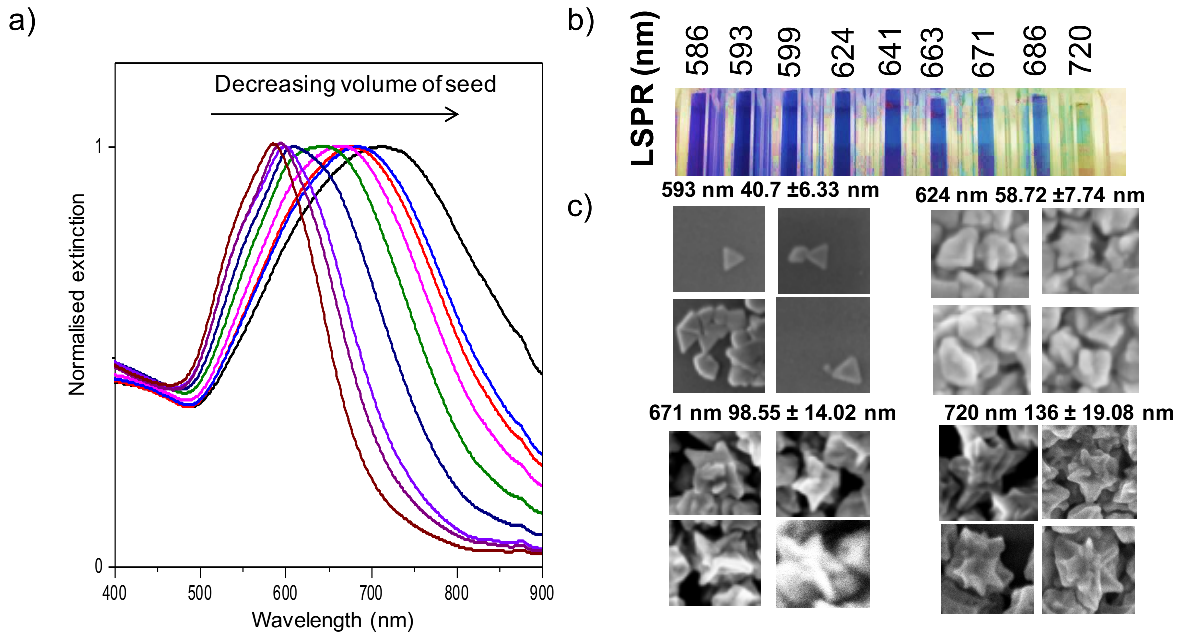

Figure S1 Normalized extinction spectra and SEM images of Au NS synthesized using different volumes of Au multi-twinned seeds.

By decreasing the volume of gold multi-twinned seed added to growth solution, the LSPR of the synthesized NS are red shifted as seen in the extinction spectra and NS color change. The SEMs of 4 NS demonstrate the size and shape of synthesized particles associated with the LSPR. High volumes of seed (500 μL) yield triangular shaped particles, whilst decreasing the volume allows more branch growth to occur and the synthesized particles are star shaped (10 μL and 1 μL).

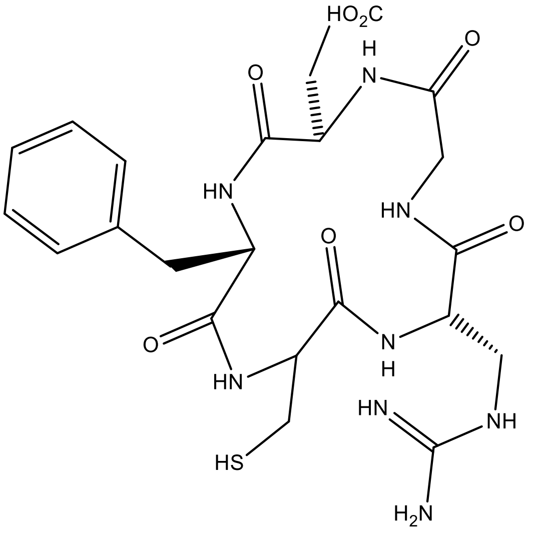

Figure S2 Structure of Cyclic(-Arg-Gly-Asp-D-Phe-Cys)

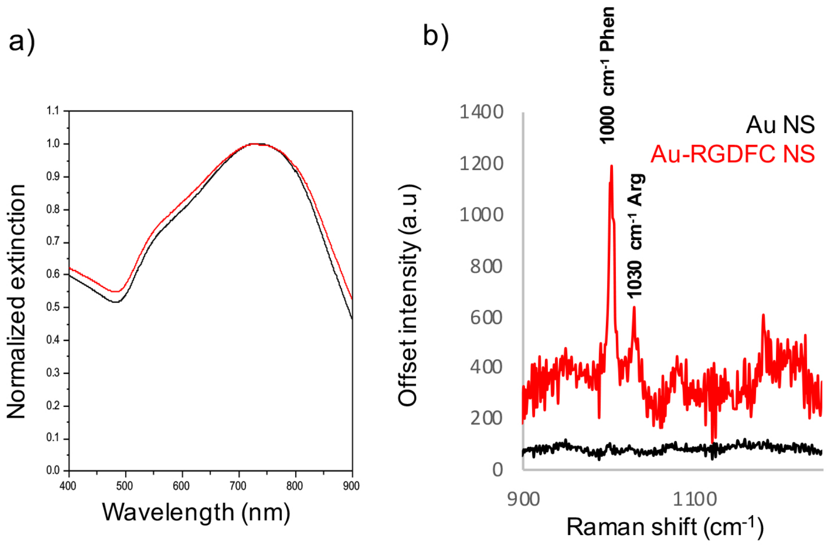

Figure S3 Normalized extinction spectrum of Au NS (black) and Au-RGDFC NS (red)and b) SERS spectra of Au NS (black) and Au-RGDFC NS (red) for NS. The SERS spectra are average from 10 nanoparticle clusters in the focus of a 50x microscope objective, excited using 0.8 mW of 633 nm laser excitation and centered at 1000 cm^{-1}.

Cyclic RGDFC was successfully synthesized to both NS evident in the blue shifting and peak broadening which occurred in the extinction spectra. The SERS spectra also indicate conjugation has occurred due to the presence of the 1000 cm^{-1} peak from the phenylalanine and 1030 cm^{-1} arginine residues of the RGDFC molecules.

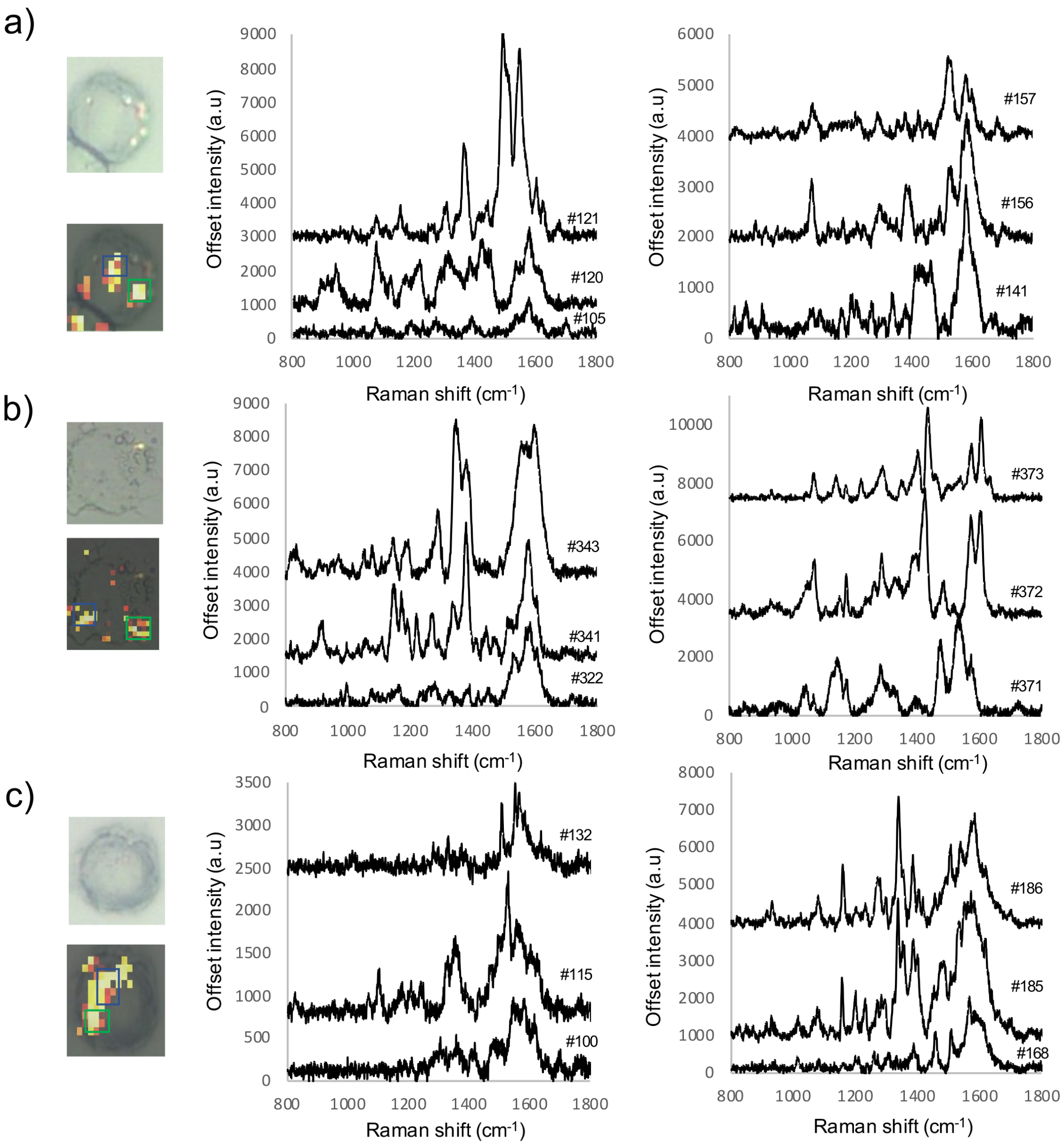

Figure S4 False color images of SW620 cell incubated with Au-RGDFC NS created using the MCR generated spectrum of purified $\alpha_v\beta_3$ integrin as a reference. MCR reference spectrum and extracted spectra from within the blue box and green box for each cell. Cells were mapped using a 50x microscope objective, 1x1 µm step size, 633 nm laser excitation with a laser power of 4 mW, scanning between 800-2000 cm $^{-1}$.

Figure S4 demonstrates the reproducibility of the signal obtained from cells incubated with Au-RGDFC NS. Of the 3 cells presented, the DCLS false color images indicate many areas where the spectra have similar spectral contributions as the MCR $\alpha_v\beta_3$ spectrum. The signal is also concentrated to small areas of the cell due to integrin clustering that occurs on the cell membrane.

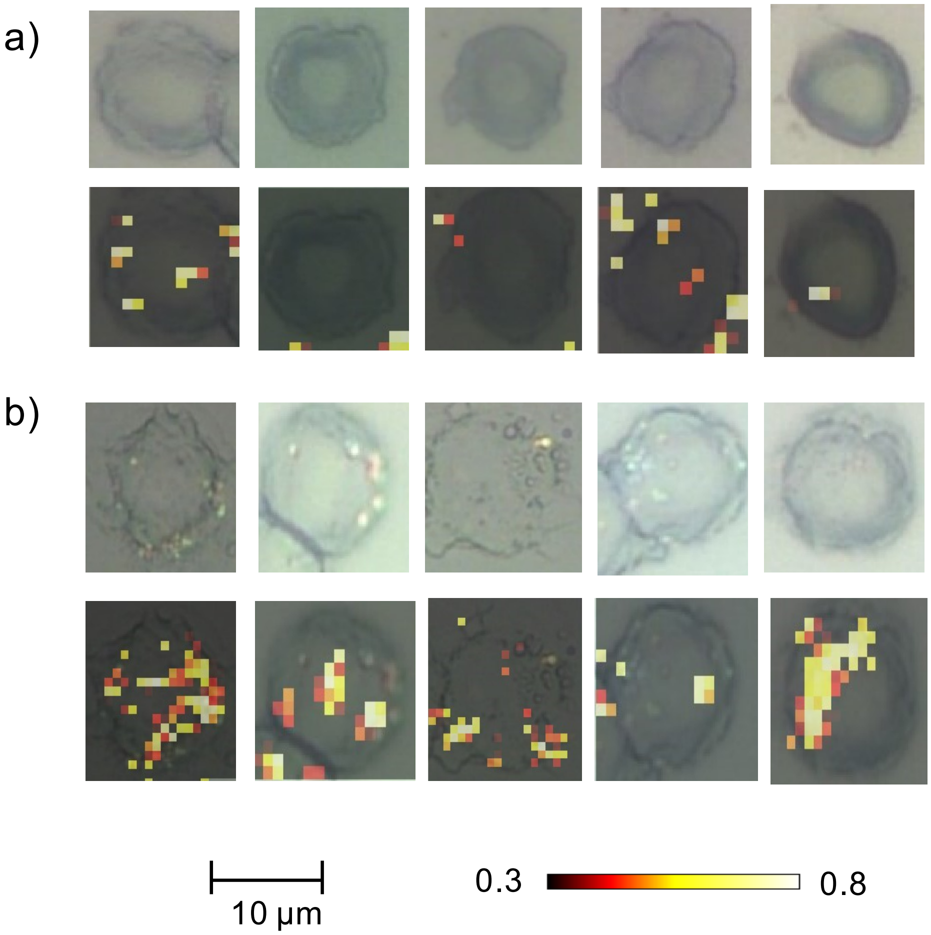

Figure S5 Comparison of white light and DCLS false color images obtained from 5 cells that were incubated with a) Au NS and b) Au-RGDFC NS. Cells were mapped using a 50x microscope objective, 1x1 µm step size, 633 nm laser excitation with a laser power of 4 mW, scanning between 800-2000 cm $^{-1}$

By comparing the DCLS false color images of cells incubated with Au and Au-RGDFC NS, there is a clear difference in the intensity and number of the pixels in each map. This is due to the Au-RGDFC NS correctly targeting the $\alpha_v\beta_3$ integrin and enhancing it and other cellular components, whereas the Au NS are not attracted to the $\alpha_v\beta_3$ and the small number of spectra with signal is due to NS electrostatically binding to the cell surface, while most are washed away or stuck to the glass surface, enhancing the protein corona.

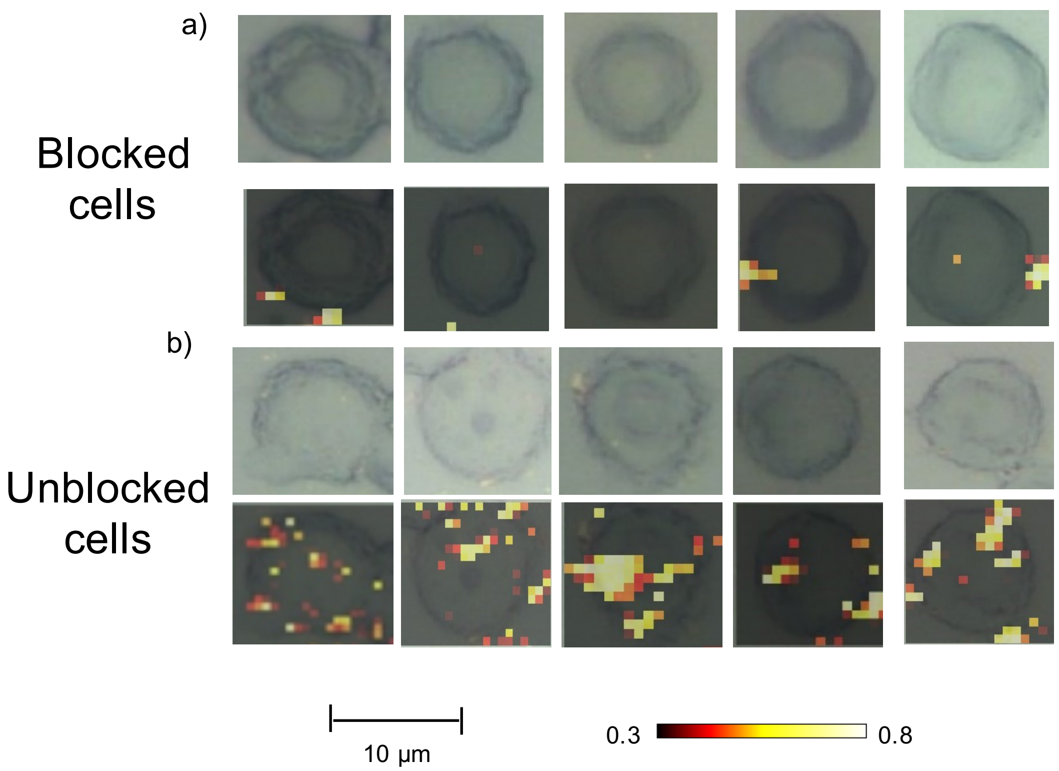

Figure S6 Comparison of white light and DCLS false color images obtained from 5 cells that were a) blocked with RGD and incubated with Au-RGDFC NS and b) unblocked cells incubated with Au-RGDFC NS Cells were mapped using a 50x microscope objective, 1x1 µm step size, 633 nm laser excitation with a laser power of 4 mW, scanning between 800-2000 cm $^{-1}$.

Comparing the DCLS false color images of blocked and unblocked cells there is decrease in the number of spectra with similar spectral components as $\alpha_v\beta_3$ integrin when the cells were blocked with RGD as there are a smaller number of $\alpha_v\beta_3$ integrin binding sites available for Au-RGDFC NS.

Table S1 $\alpha_v\beta_3$ integrin peak assignments

SERS band (cm^{-1})	Assignment
926	ν(COO^-)
956	Leucine
1000, 1207	Phenylalanine
1030	Arginine
1078	Lysine
1153	C-N protein
1233	Amide II
1288,1446	C-H bending
1386	Tryptophan
1507-1515	C-C, C=C
1586-1591, 1625	Phenylalanine, tryptophan, C-C

References

(1) Bauer, K.M., Lambert, P.A., Hummon, A.B,. *Proteomics*, 2012, **2012**, 1928-1937
(2) Xiao, L.; Bailey, K.A.; Wang, H.; Schultz. Z.D. *Anal. Chem.*, **2017**, *89* (17), pp 9091–9099
(3) Xiao, L.; Wang, H.; Schultz. Z.D *Anal. Chem.*, **2016**, *88* (12), pp 6547–6553
(4) Portela, A.; Yano, T.; Santschi, C.; Martin, O.J.F.; Tabata, H.; Hara, M. *J. Biophotonics,* **2017** 10(2) pp 294-302
